# Phylogenetic analysis of the viral proteins VP4/VP7 of circulating human rotavirus strains in China from 2016 to 2019 and comparison of their antigenic epitopes with those of vaccine strains

**DOI:** 10.3389/fcimb.2022.927490

**Published:** 2022-08-08

**Authors:** Tongyao Mao, Mengxuan Wang, Jindong Wang, Yalin Ma, Xiafei Liu, Mingwen Wang, Xiaoman Sun, Lili Li, Huiying Li, Qing Zhang, Dandi Li, Zhaojun Duan

**Affiliations:** ^1^ NHC Key Laboratory for Medical Virology and Viral Diseases, National Institute for Viral Disease Control and Prevention, Chinese Center for Disease Control and Prevention, Beijing, China; ^2^ Department of Medical Microbiology, Weifang Medical University, Weifang, China; ^3^ School of Public Health, Gansu University of Chinese Medicine, Lanzhou, China

**Keywords:** rotavirus vaccines, antigenic epitopes, diarrhea, VP7, VP4, China

## Abstract

Group A rotaviruses (RVAs) are the most common etiological agents of severe acute diarrhea among children under 5 years old worldwide. At present, two live-attenuated RVA vaccines, LLR (G10P[15]) and RotaTeq (G1–G4, G6 P[8], P[5]), have been introduced to mainland China. Although RVA vaccines can provide homotypic and partially heterotypic protection against several strains, it is necessary to explore the genetic and antigenic variations between circulating RVAs and vaccine strains. In this study, we sequenced viral protein VP7 and VP4 outer capsid proteins of 50 RVA strains circulating in China from 2016 to 2019. The VP7 and VP4 sequences of almost all strains showed high homology to those of previously reported human strains and vaccine strains of the same genotype. However, in the presumed antigenic epitopes of the VP7 and VP4, multiple amino acid variations were found, regardless of the G and P genotypes of these strains. Moreover, all circulating G3 RVA strains in China potentially possess an extra N-linked glycosylation site compared with the G3 strain of RotaTeq. The potential N-linked glycosylation site at residues 69–71 was found in all G9 strains in China but not in the G9 strain of the Rotavac or Rotasill vaccine. These variations in antigenic sites might result in the selection of strains that escape the RVA neutralizing-antibody pressure imposed by vaccines. Furthermore, the G4 and P[6] genotypes in this study showed high homology to those of porcine strains, indicating the transmission of G4 and P[6] genotypes from pigs to humans in China. More genetic surveillance with antigenic evaluation in prevalent RVAs is necessary for developing and implementing rotavirus vaccines in China.

## Introduction

Group A rotaviruses (RVAs) were first detected in the 1970s and are the most common etiological agents of severe acute gastroenteritis among children under 5 years old worldwide (Banyai et al., 2018; Raju et al., 2019). Rotavirus gastroenteritis (RVGE) caused an estimated 128,500 deaths in 2016, mostly in low-income countries, and rotavirus vaccines have reduced deaths by more than 280,000 (Troeger et al., 2018). In Asia, rotavirus accounts for 37.5% of hospitalized gastroenteritis cases (Troeger et al., 2018). In addition, treatment of RVGE incurs the greatest cost (US$365 million annually) in China, followed by Japan and India (Kawai et al., 2012). Vaccination is the most effective means of preventing RVGE. The first (RotaTeq and Rotarix, approved in 2008 and 2009, respectively) and subsequent (Rotavac and Rotasill, approved in 2018) rotavirus vaccines were prequalified by the World Health Organization (World Health Organization, 2021; Sadiq et al., 2022) and have contributed to the rapid decline in deaths caused by RVAs. The global rates of diarrhea among hospitalized children under 5 years old before the introduction of rotavirus vaccines in 2013 and after the introduction of rotavirus vaccines in 2019 were 38% and 23%, respectively (Sadiq et al., 2018; World Health Organization, 2021).

RVAs are segmented double-stranded RNA viruses with high genetic diversity. The 11 RNA segments of RVAs encode six structural viral proteins (VP1–4, 6, and 7) and six non-structural proteins (NSP1–6) (Ramig, 1997). The most common classification system for RVAs is based on the VP7 and VP4 genes, i.e., the G/P genotype. G1P[8], G2P[4], G3P[8], G4P[8], and G9P[8] are the most prevalent RVA genotypes worldwide (Sadiq et al., 2018). G9P[8] has become predominant in China in the last decade. G12P[6], G12P[8], and G8P[8] are emerging worldwide but are rarely reported in China (Sadiq et al., 2018).

VP7 and VP4 are outer capsid proteins of RVA, and their genes are the most important antigenic genes for the major neutralizing antigenic epitopes in RVA vaccines (Estes and Cohen, 1989). However, the segmented genomic complexity with evolution of the VP7 and VP4 genes has reduced the efficacy of RVA vaccines (Estes and Cohen, 1989). We performed phylogenetic analysis of the VP4 and VP7 of circulating RVA strains in China from 2016 to 2019 and identified potentially important antigenic disparities compared with these proteins of vaccine strains, to facilitate the development and introduction of RVA vaccines in China.

## Materials and methods

### Sample selection

A total of 50 rotavirus strains of seven genotypes [G1P[8] (n = 6), G2P[4] (n = 8), G2P[8] (n = 1), G3P[8] (n = 13), G4P[6] (n = 1), G9P[8] (n = 20), and G3P[X] (n = 1)] were included in this study. These strains were selected based on preliminary phylogenetic analyses of partial sequences of the VP7 and VP4 genes (146–682 nucleotides each) (Gouvea et al., 1990; Asmah et al., 2001; Iturriza et al., 2004). These rotavirus genotypes represent those circulating in mainland China from January 2016 to December 2019. The strains were collected and stored in our laboratory, and information on these strains is listed in **Table 1**. Subgenotypic lineages were assigned as described previously (Gouvea et al., 1990; Asmah et al., 2001; Iturriza et al., 2004). The nucleotide sequences of VP7 (sequence ID: HM800948) and VP4 (sequence ID: JQ013506) of the LLR strains were obtained from GenBank. The nucleotide sequences of VP7 and VP4 of the vaccines [RotaTeq (G1–G4, G6 P[8], P[5]), Rotarix (G1P[8]), Rotavac (G9P[11]), and Rotasill (G9P[X])] are published elsewhere (Zeller et al., 2012; Genggeng et al., 2021).

**Table 1:** VP7 and VP4 lineage, date of isolation, and patient information for the Chinese rotavirus strains analyzed in this study and vaccine strains.

Strain	G-genotype/lineage	P-genotype/lineage	Date of isolation(yy/mm)	Age(years)	GenBank identification	
					VP7	VP4
RVA/Vaccine/USA/Rotarix-A41CB052A/1988/G1P1A[8]	II	I			JN849114.1	JN849113
RVA/Vaccine/USA/RotaTeq-WI79-9/1992/G1P7[5]	III	NA			GU565057	GU565055
**RVA/Human-wt/CHN/SC18-1073/2018/G1P[8]**	I	III	18/3	<5	OM920789	OM920790
**RVA/Human-wt/CHN/SC19-1047/2019/G1P[8]**	I	III	19/3	<5	OM920745	OM920746
**RVA/Human-wt/CHN/GS16-2068/2016/G1P[8]**	I	III	16/2	<5	OM920761	OM920762
**RVA/Human-wt/CHN/JL18-1009/2018/G1P[8]**	I	III	18/1	<5	OM920777	OM920778
**RVA/Human-wt/CHN/SD17-0275/2017/G1P[8]**	I	III	17/11	<5	OM920791	OM920792
**RVA/Human-wt/CHN/SZ16-2007/2016/G1P[8]**	I	III	16/1	<5	OM920799	OM920800
RVA/Vaccine/USA/RotaTeq-SC2-9/1992/G2P7[5]	II	NA			GU565068	GU565066
**RVA/Human-wt/CHN/SD17-0050/2017/G2P[4]**	I	III	17/3	<5	OM920739	OM920740
**RVA/Human-wt/CHN/SD16-0334/2016/G2P[4]**	IV	III	16/12	<5	OM920737	OM920738
**RVA/Human-wt/CHN/AH17-2033/2017/G2P[4]**	IV	III	17/3	<5	OM920731	OM920732
**RVA/Human-wt/CHN/SC17-1038/2017/G2P[8]**	IV	III	17/3	<5	OM920785	OM920786
**RVA/Human-wt/CHN/GX19-1005/2019/G2P[4]**	IV	III	19/1	<5	OM920727	OM920728
**RVA/Human-wt/CHN/HEB16-1258/2018/G2P[4]**	IV	III	19/-	<5	OM920735	OM920736
**RVA/Human-wt/CHN/FJ16-1017/2016/G2P[4]**	IV	III	16/1	<5	OM920733	OM920734
**RVA/Human-wt/CHN/JL19-1276/2019/G2P[4]**	IV	III	19/5	<5	OM920729	OM920730
**RVA/Human-wt/CHN/SZ16-2064/2016/G2P[4]**	IV	III	16/3	<5	OM920741	OM920742
RVA/Vaccine/USA/RotaTeq-WI78-8/1992/G3P7[5]	II	NA			GU565079	GU565077
**RVA/Human-wt/CHN/GX18-1008/2018/G3P[8]**	V	III	18/2	<5	OM920769	OM920770
**RVA/Human-wt/CHN/SZ18-2040/2018/G3P[8]**	V	III	18/1	<5	OM920805	OM920806
**RVA/Human-wt/CHN/GX19-1019/2019/G3P[8]**	V	III	19/3	<5	OM920753	OM920754
**RVA/Human-wt/CHN/SC19-1170/2019/G3**P[X]	IV	NA	19/11	<5	OM920812	–
**RVA/Human-wt/CHN/SZ16-2066/2016/G3P[8]**	I	III	16/3	<5	OM920803	OM920804
**RVA/Human-wt/CHN/FJ16-1342/2016/G3P[8]**	I	III	16/12	<5	OM037833	OM037828
**RVA/Human-wt/CHN/JL18-1081/2018/G3P[8]**	I	III	18/2	<5	OM038053	OM038048
**RVA/Human-wt/CHN/GX16-1148/2016/G3P[8]**	I	III	16/5	<5	OM920767	OM920768
**RVA/Human-wt/CHN/GD18-2005/2018/G3P[8]**	I	III	17/2	<5	OM038086	OM038081
**RVA/Human-wt/CHN/HN19-V1048/2019/G3P[8]**	I	III	19/4	<5	OM920757	OM920758
**RVA/Human-wt/CHN/HN17-2170/2017/G3P[8]**	I	III	17/11	<5	OM920775	OM920776
**RVA/Human-wt/CHN/SC16-1029/2016/G3P[8]**	I	III	16/1	<5	OM037943	OM037938
**RVA/Human-wt/CHN/NM16-1108/2016/G3P[8]**	I	III	16/5	<5	OM037910	OM037905
**RVA/Human-wt/CHN/HEB16-1045/2016/G3P[8]**	I	III	16/1	<5	OM037877	OM037872
RVA/Vaccine/USA/RotaTeq-BrB-9/1996/G4P7[5]	II	NA			GU565090	GU565088
**RVA/Human-wt/CHN/SZ18-2049/2018/G4P[6]**	I	NA	18/2	<5	OM920725	OM920726
RVA/Vaccine/IND/Rotavac-116E/AG/G9P[11]	II	NA			FJ361209	FJ361204
RVA/Vaccine/USA/RotaTeq-WI79-4/1992/G6P1A[8]	-	II			GU565046	GU565044
RVA/Vaccine/CHN/LLR/1985/G10P[12]	-	NA			HM800948	JQ013506
**RVA/Human-wt/CHN/SD18-0052/2018/G9P[8]**	VI-e	III	18/2	<5	OM920793	OM920794
**RVA/Human-wt/CHN/SH18-1018/2018/G9P[8]**	VI-e	III	18/2	<5	OM920797	OM920798
**RVA/Human-wt/CHN/NM17-1062/2017/G9P[8]**	VI-e	III	17/3	<5	OM920783	OM920784
**RVA/Human-wt/CHN/GS16-2114/2016/G9P[8]**	VI-e	III	16/4	<5	OM920763	OM920764
**RVA/Human-wt/CHN/JL19-1034/2019/G9P[8]**	VI-e	III	19/1	<5	OM920755	OM920756
**RVA/Human-wt/CHN/HN17-2004/2017/G9P[8]**	VI-e	III	17/1	<5	OM920773	OM920774
**RVA/Human-wt/CHN/NM16-1059/2016/G9P[8]**	VI-e	III	16/3	<5	OM920781	OM920782
**RVA/Human-wt/CHN/GX16-1119/2016/G9P[8]**	VI-e	III	16/3	<5	OM920765	OM920766
**RVA/Human-wt/CHN/SH19-1061/2019/G9P[8]**	VI-e	III	19/3	<5	OM920749	OM920750
**RVA/Human-wt/CHN/SD18-0121/2018/G9P[8]**	VI-e	IV	18/3	<5	OM920795	OM920796
**RVA/Human-wt/CHN/HEB16-1059/2016/G9P[8]**	VI-e	III	16/1	<5	OM920771	OM920772
**RVA/Human-wt/CHN/SZ16-2019/2016/G9P[8]**	VI-e	III	16/1	<5	OM920801	OM920802
**RVA/Human-wt/CHN/SZ18-2195/2018/G9P[8]**	VI-e	III	18/11	<5	OM920807	OM920808
**RVA/Human-wt/CHN/SC18-1013/2018/G9P[8]**	VI-e	III	18/1	<5	OM920787	OM920788
**RVA/Human-wt/CHN/GD18-2017/2018/G9P[8]**	VI-e	III	18/1	<5	OM920759	OM920760
**RVA/Human-wt/CHN/HN19-V1057/2019/G9P[8]**	VI-e	III	19/5	<5	OM920743	OM920744
**RVA/Human-wt/CHN/JL18-1318/2018/G9P[8]**	VI-e	III	18/11	<5	OM920779	OM920780
**RVA/Human-wt/CHN/SZ18-2197/2018/G9P[8]**	III-d	III	18/12	<5	OM920809	OM920810
**RVA/Human-wt/CHN/GX19-1013/2019/G9P[8]**	III-d	III	19/3	<5	OM920751	OM920752
**RVA/Human-wt/CHN/SC19-1063/2019/G9P[8]**	III-d	III	19/4	<5	OM920747	OM920748

### Nucleic acid extraction

Stool samples (100 mg) were suspended in 1 ml of Hanks’ Balanced Salt Solution (HBSS) solution, homogenized by vortex mixing, and centrifuged. Viral RNA was extracted from stool samples and purified using the QIAamp viral RNA Mini Kit (Qiagen, Hilden Germany) according to the manufacturer’s instructions.

### Amplification of VP7 and VP4

Extracted viral double-stranded RNA was used as the template for Multiplex RT-PCR using specific VP7 and VP4 consensus primer pairs (Matthijnssens et al., 2006). The full CDS region of VP7 (978 base pairs) and partial CDS region of VP4 (2088 base pairs) were amplified. RT-PCR was performed using the Qiagen One-Step RT-PCR kit (Qiagen, Hilden Germany) according to the manufacturer’s instructions. RT-PCR was also performed with an independent RT step using the SuperScript™ III Reverse Transcription Kit (Invitrogen California, USA). Taq polymerase was activated for 3 min at 94°C, followed by 35 cycles of amplification (30 s at 94°C, 30 s at 55°C, and 60 s at 72°C), with a final extension for 10 min at 72°C.

### Sequence analysis

The nucleotide sequences of VP7 and VP4 were analyzed, and consensus alignments were conducted using ClustalW (Larkin et al., 2007). Maximum-likelihood phylogenetic trees were drawn in MEGA 7.0 software based on the GTR+G+I model and calculated by the bootstrap method using 1,000 replicates. All positions with less than 95% site coverage were eliminated. Amino acid sequence similarity was calculated using MegAlign (DNA Star), and potential N-linked glycosylation sites were screened using NetNGlyc1.0 Server (https://services.healthtech.dtu.dk/service.php?NetNGlyc-1.0). For antigenic characterization, sequences were aligned using BioEdit, and structural diagrams were generated using Pymol 2.5. The VP7 and VP4 sequences were submitted to GenBank under accession numbers OM920725–920812 (sequenced in this study) or downloaded (OM037825–038088).

## Results

### Phylogenetic and sequence analyses of the VP7 gene

**Figure 1:**
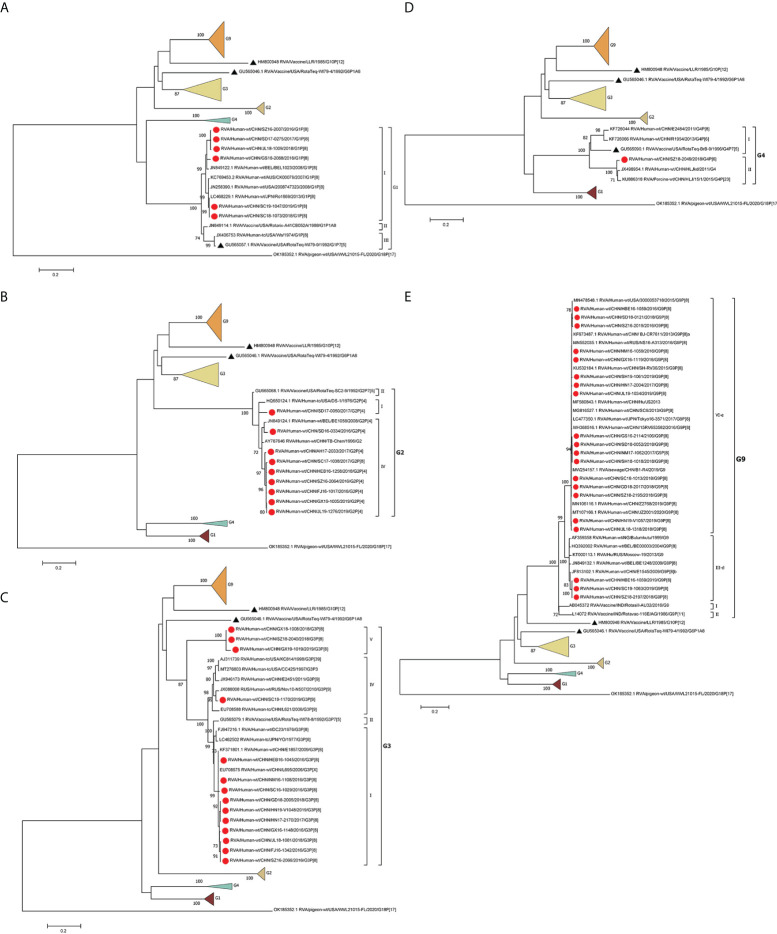
Phylogenetic analysis of the VP7 proteins of representative RVA strains and vaccine strains (RotaTeq G1–4, G6, Rotarix G1, LLR, Rotasill G9, and Rotavac G9). Maximum-likelihood **(A–E)** trees were constructed based on the complete VP7 CDS region gene sequences (978 base pairs). A GTR+G+I nucleotide substitution model was used to construct the phylogenetic tree. The pigeon RVA strain WVL21015-FL was used as the outgroup. Chinese strains are marked by red dots and vaccine strains by black triangles. Bootstrap values (1,000 replicates) of 70% are shown.

The phylogenetic analysis showed that the G1 strains (n = 6) clustered in G1 lineage I (**Figure 1A**) with strains from Belgium, the USA, Australia, and Japan. These strains showed 93.7%–95.3% amino acid sequence identities with Rotarix G1 (G1 lineage II) and 92.7%–94.3% with RotaTeq G1 (G1 lineage III) (**Table 2**). Eight G2 strains were of G2 lineage IV, and one was G2 lineage I (**Figure 1B**). The eight G2 strains clustered with USA and Belgian G2 strains. These G2 strains showed 94.3%–95.7% amino acid identity with RotaTeq G2 (G2 lineage II) (**Table 2**).

**Table 2:** Distance matrix for VP7 and VP4 based on amino acid identities.

Strain	G-genotype/lineage	G1 of Rotarix	G1 of RotaTeq	G2 of RotaTeq	G3 of RotaTeq	G4 of RotaTeq	G6 of RotaTeq	G9 of Rotavac	G10 of LLR	G9 of Rotasill	P-genotype/lineage	P[8] of Rotarix	P[8] of RotaTeq	P[5] of RotaTeq	P[11] of Rotavac	P[12] of LLR
**SC18-1073**	G1/I	95.3	94.3	74.7	80	75	80.3	78.7	77.0	77.3	P[8]/3	95.4	96.2	74.7	67.2	78.2
**SC19-1047**	G1/I	95.3	94.3	74.7	80	75	80.3	78.7	77.0	77.3	P[8]/3	94.8	95.2	75.1	67.2	78.5
**GS16-2068**	G1/I	94.3	92.7	73.7	79	75	80.7	77.3	76.3	76.3	P[8]/3	95.2	96.4	75.1	66.7	78.5
**JL18-1009**	G1/I	94	93	74.3	79.7	74.7	80.7	77.3	77.3	76.7	P[8]/3	95.2	96.4	75.1	66.7	78.5
**SD17-0275**	G1/I	93.7	92.7	74	79.3	74.3	80.3	77	77.0	76.3	P[8]/3	95.2	96.4	75.1	66.7	78.5
**SZ16-2007**	G1/I	93.7	93.3	74	79.3	74.3	80.3	77	77.0	76.3	P[8]/3	95.2	95.4	74.7	67.2	78.5
**SD17-0050**	G1/I	73.2	73.2	95.7	71.9	67.3	73.2	74.2	71.2	75.9	P[4]/3	88.2	89.2	68.6	57.9	71.3
**SD16-0334**	G2/IV	73.6	74.2	95	73.9	67.3	74.2	75.3	72.6	75.3	P[4]/3	89.2	90.2	68.7	57.2	71.8
**AH17-2033**	G2/IV	73.2	73.9	95.3	73.6	67.7	73.9	74.9	71.9	75.3	P[4]/3	88.6	89.7	68.4	57.8	71.8
**SC17-1038**	G2/IV	73.2	73.6	95.3	73.2	67.7	73.6	74.6	71.6	75.6	P[8]/3	95	95.8	74.3	66.9	77.4
**GX19-1005**	G2/IV	73.2	73.6	95	73.2	67.7	73.6	74.6	71.6	75.6	P[4]/3	88.5	89.5	68.6	58	71.4
**HEB16-1258**	G2/IV	73.2	73.6	95	73.2	67.7	73.6	74.6	71.6	75.6	P[4]/3	88.6	89.6	68.4	58	71.6
**FJ16-1017**	G2/IV	73.2	73.6	95	73.2	67.7	73.6	74.6	71.6	75.6	P[4]/3	88.2	89.2	68.3	57.8	71.1
**JL19-1276**	G2/IV	73.2	73.6	95	73.2	67.7	73.6	74.6	71.7	75.6	P[4]/3	88.5	89.5	68.6	58	71.4
**SZ16-2064**	G2/IV	73.6	73.9	94.6	73.6	67.3	73.9	74.2	71.9	75.3	P[4]/3	88.3	89.3	68.4	58	71.4
**GX18-1008**	G3/V	81	80.2	76	92.7	80.2	87.8	86.6	85.9	87.8	P[8]/3	95.2	96	74.7	66.9	77.6
**SZ18-2040**	G3/V	81	80.2	76	92.7	80.2	87.8	86.6	85.9	87.8	P[8]/3	95.2	96	74.5	66.9	77.6
**SD17-0287**	G3/V	79.8	79.1	74.9	91.6	78.6	86.3	85.5	84.8	86.3	P[8]/3	95.2	96	74.5	66.7	78
**GX19-1019**	G3/V	79.5	79.8	76.4	92	78.2	86.6	84	83.7	85.5	P[8]/3	95.4	96.2	74.5	66.7	77.8
SC19-1170	G3/IV	81.4	82.5	75.7	93.9	78.6	86.3	83.2	83.7	84	/	/	/	/	/	/
**SZ16-2066**	G3/I	81.4	81.7	75.7	96.9	77.5	85.1	83.2	83.7	84.4	P[8]/3	95.2	95.6	75.1	66.9	78.5
**FJ16-1342**	G3/I	81.4	81.7	75.7	96.9	77.5	85.1	83.2	83.7	84.4	P[8]/3	95.6	96	75.3	66.9	78.7
**JL18-1081**	G3/I	81	82.1	76	96.6	77.1	84.7	82.8	83.7	84	P[8]/3	95.6	96.4	74.7	66.9	78
**GX16-1148**	G3/I	81.4	81.7	75.7	97.3	77.5	85.1	82.8	83.7	84.4	P[8]/3	95.2	96	74.7	66.5	77.6
**GD18-2005**	G3/I	81.4	81.7	75.7	96.9	77.5	85.1	83.2	83.7	84.4	P[8]/3	95.4	95.8	75.3	67.2	78.7
**HN19-V1048**	G3/I	81.4	81.7	75.7	96.9	77.5	85.1	83.2	83.7	84.4	P[8]/3	95.4	95.8	75.3	67.2	78.7
**HN17-2170**	G3/I	81.4	81.7	75.7	96.9	77.5	85.1	83.2	83.7	84.4	P[8]/3	95.2	95.6	75.3	67.2	78.7
**SC16-1029**	G3/I	81.4	81.7	76	96.6	77.5	85.1	82.8	83.7	84	P[8]/3	95.2	95.6	75.3	67.4	78.5
**NM16-1108**	G3/I	81.4	81.7	75.7	96.9	77.5	85.1	83.2	83.7	84.4	P[8]/3	95.4	95.8	74.5	66.9	78.2
**HEB16-1045**	G3/I	81	81.4	75.3	96.6	77.1	84.7	82.8	83.3	84	P[8]/3	95.4	96.2	74.9	67.2	78.7
**SZ18-2049**	GIV/I	78.1	76.9	71.3	75.9	87.3	75	77.5	75.9	76.5	P[6]/NA	78.9	78.4	70.3	55.3	75.2
**SD18-0052**	G9/VI-e	80.5	79.9	78.2	80.7	79.2	83.2	93.6	81.5	94.3	P[8]/3	95.6	96.4	74.7	66.9	78
**SH18-1018**	G9/VI-e	80.5	79.9	78.2	80.7	79.2	83.2	93.6	81.5	94.3	P[8]/3	95.6	96.4	74.7	66.9	78
**NM17-1062**	G9/VI-e	80.5	79.9	78.2	80.7	79.2	83.2	93.6	81.5	94.3	P[8]/3	95.6	96.4	74.7	66.9	78
**GS16-2114**	G9/VI-e	80.5	79.9	78.2	80.7	79.2	83.2	93.6	81.5	94.3	P[8]/3	95.6	96.4	74.7	66.9	78
**JL19-1034**	G9/VI-e	80.5	79.9	78.2	80.7	79.2	83.2	93.6	81.5	94.3	P[8]/3	95.6	96.4	74.7	66.9	78
**HN17-2004**	G9/VI-e	80.2	79.5	77.9	80.7	78.9	83.2	93.3	81.5	94	P[8]/3	95.2	96	74.3	66.7	78
**NM16-1059**	G9/VI-e	80.5	79.9	78.2	80.7	79.2	83.2	93.6	81.5	94.3	P[8]/3	95.2	96	74.7	66.9	78.5
**GX16-1119**	G9/VI-e	80.5	79.9	78.2	80.7	79.2	83.2	93.6	81.5	94.3	3	95.2	96	74.7	66.9	78.5
**SH19-1061**	G9/VI-e	80.5	79.9	78.2	80.7	79.2	83.2	93.6	81.5	94.3	3	95.6	96.4	74.7	66.9	78
**SD18-0121**	G9/VI-e	80.5	79.9	78.6	80.3	79.2	82.9	93.3	81.2	94	4	94.8	93.9	74.7	66.9	78
**HEB16-1059**	G9/VI-e	80.5	79.9	78.6	80.3	79.2	82.9	93.3	81.2	94	3	94.8	95.4	74.1	66.3	77.8
**SZ16-2019**	G9/VI-e	80.5	79.9	78.6	80.3	79.2	82.9	93.3	81.2	94	3	95.4	96.2	74.9	66.7	77.8
**SZ18-2195**	G9/VI-e	80.2	79.5	78.2	80.3	78.9	82.9	93.3	81.2	94	3	95.6	96.4	74.7	66.9	78
**SC18-1013**	G9/VI-e	79.5	79.9	78.2	80.7	79.2	83.2	93.6	81.5	94.3	3	95.6	96.4	74.7	66.9	78
**GD18-2017**	G9/VI-e	80.2	79.5	78.2	80.3	78.9	82.9	93.6	81.2	94	3	95.4	96.2	74.7	66.7	78
**HN19-V1057**	G9/VI-e	80.5	79.9	78.2	80.7	79.2	83.2	93.6	81.5	94.3	3	95.6	96.4	74.7	66.9	78
**JL18-1318**	G9/VI-e	80.2	79.5	77.9	80.3	78.9	82.9	93.6	81.2	94	3	95.6	96.4	74.7	66.9	78
**SZ18-2197**	G9/III-d	79.5	79.5	77.6	81	78.5	82.6	91.9	82.2	93.3	3	95.2	95.8	74.7	67.2	78
**GX19-1013**	G9/III-d	80.5	79.5	77.6	80.7	78.2	82.2	91.6	81.9	93	3	95.2	95.8	74.5	66.9	77.8
**SC19-1063**	G9/III-d	79.5	79.5	77.6	81	78.5	82.6	91.9	82.2	93.3	3	95.2	95.8	74.5	66.9	77.8

Intragenotype similarities with Rotarix and RotaTeq are in orange and blue, respectively.

The circulating G3 strains were included in G3 lineages I, IV, and V and clustered with strains from Belgium, the USA, Russia, Japan, and G3 strains isolated previously in China (**Figure 1C**). RotaTeq G3 had higher amino acid homology (96.6%–97.3%) with G3 lineage I than with G3 lineage IV (93.9%) or G3 lineage V (91.6%–92.7%) (**Table 2**). G4 is rare in China; the one VP4 sequence sequenced in this study showed 84.41%–85.01% homology to that of other human G4 strains reported in China and 96.84% and 95.87% identities with that of HLJKD and HLJ strains isolated from porcine stool samples in China (**Figure 1D**). This G4 strain had low pairwise homology (87.3%) to RotaTeq G4 (**Table 2**).

Seventeen G9 strains were included in G9 lineage VI-e and three in G9 lineage III-d. The 20 G9 strains clustered with strains from Belgium, Russia, and China (**Figure 1E**). They showed 91.6%–93.6% identity with the G9 strain of Rotavac (lineage II) and 93.3–94.3% identity with the G9 strain of Rotasill (lineage I) (**Table 2**).

### Phylogenetic and sequence analysis of the VP4 gene

**Figure 2:**
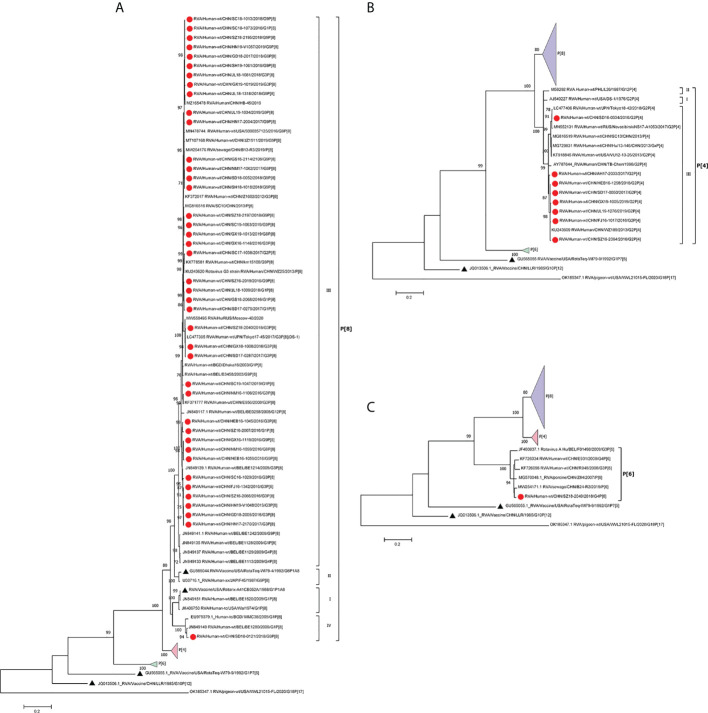
Phylogenetic analysis of the VP4 protein of circulating and vaccine RVA strains (RotaTeq P[8], P[5], Rotarix P[8], LLR). Maximum-likelihood trees **(A–C)** were constructed based on the partial VP7 CDS region sequences (2088 base pairs). A GTR+G+I nucleotide substitution model was used to construct the phylogenetic tree. The pigeon RVA strain WVL21015-FL was used as the outgroup. Chinese strains are marked by red dots and vaccine strains by black triangles. Bootstrap values (1,000 replicates) of 70% are shown.

Most P[8] strains were grouped in P[8] lineage III with P[8] strains previously isolated in China, Japan, Bangladesh, and Russia (**Figure 2**A). Only one P[8] (with G9) in 2018 belonged to P[8] lineage IV; that strain had 93.9% identity with RotaTeq P[8] (lineage I) and 94.8% with Rotarix P[8] (lineage II). The P[8] lineage III strains had 95.4%–96.4% identities with RotaTeq P[8] and 94.8%–95.6% with Rotarix P[8] (**Table 2**).

In the phylogenetic analysis, the eight P[4] strains belonged to P[4] lineage III and clustered with previous Chinese P[4] strains (**Figure 2B**). They had 88.2%–90.2% amino acid similarities to P[8] of RotaTeq and Rotarix but low similarities (68.3%–74.3%) to P[5] of RotaTeq (**Table 1**). In addition, only P[6] clustered closer to porcine P[6] strains than to previously reported human P[6] strains isolated in China (**Figure 2C**).

### Comparison of VP7 neutralizing epitopes with vaccine strains

**Figure 3:**
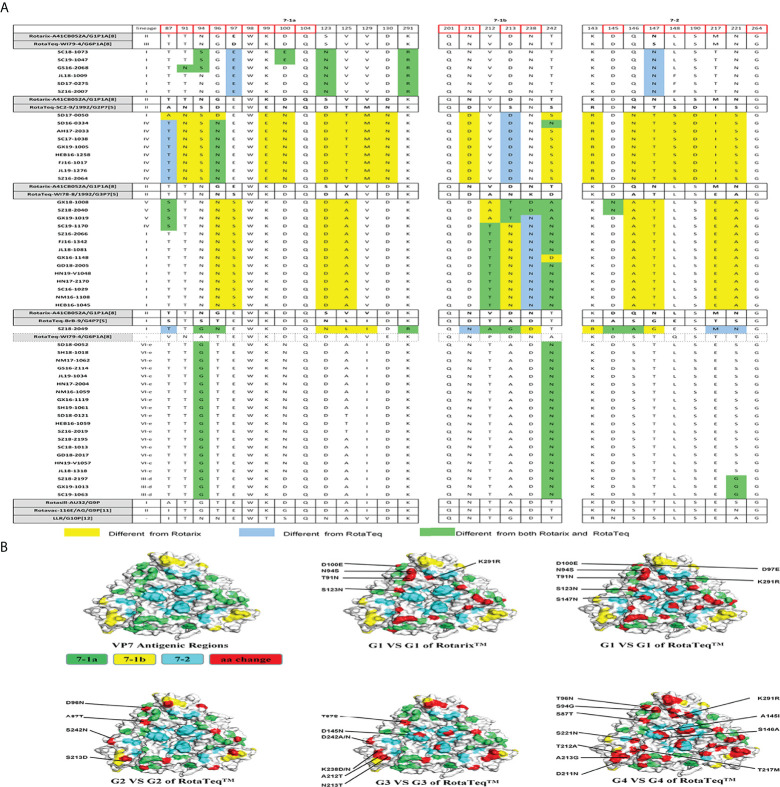
**(A)** Alignment of the antigenic epitopes in VP7 of the RVA strains circulating in China with those in Rotarix, RotaTeq, and other vaccines. Red box, the sites involved in neutralization escape. **(B)** Surface representation of the VP7 trimer (PDB 3FMG). Antigenic epitopes are in lime green (7-1a), yellow (7-1b), and cyan (7-2). Red, the surface-exposed residues that differ between circulating and vaccine RVA strains.

The VP7 protein has two critical antigenic epitopes: 7-1 (7-1a and 7-1b) and 7-2. The greatest amino acid differences were in the 7-1b subunit of VP7, followed by the 7-2 and 7-1a subunits. Of the 29 amino acid residues in VP7 neutralizing epitopes, four (W98, Q104, Q201, and G264) were conserved among all strains prevalent in China and those used in vaccines (**Figure 3A**).

G1 strains showed five residue differences from Rotarix G1 and seven from RotaTeq G1 in the VP7 antigenic epitopes (**Figure 3A**). These sites were mainly in epitope 7-1a. Several sites exhibited features of escape mutants. Compared with Rotarix G1, half of the G1 strains had N94S, and all had S123N and K291R, in the 7-1a region (**Figure 3A**). Compared with RotaTeq G1, all strains had D97E and S147N in the 7-1a and 7-2 regions (**Figure 3B**), respectively. Moreover, T91N, N94S, and D100E occurred in some G1 strains.

The G2 strains showed four amino acid differences compared with RotaTeq G2. Among them, A87T, D96N, and S213D (7-1a and 7-1b regions) might induce immunogenicity changes in the vaccine. In addition, strain SD17-0050 contained only S213D (**Figure 3A**), which was exposed at the edge of the VP7 trimer (**Figure 3B**).

Compared with RotaTeq G3, the G3 strains showed five or six amino acid differences in the VP7 antigenic epitopes. Indeed, their VP7 antigenic epitopes contained mutations at residues 238 (K238D/N) and 242 (D242A/N), both in the 7-1b epitope (**Figure 3A**). Interestingly, each of these G3 strains contained K238N or D145N, which provides a potential N-linked glycosylation site that does not exist at the corresponding position in RotaTeq G3 (Ben Hadj Zeller et al., 2012; Fredj et al., 2013). KN238 or D145N was surrounded by other residues of epitope regions (**Figure 3B**). This glycosylation may have a profound impact on the antigenicity of this epitope.

The VP7 trimer surface of G4 strain SZ18-2049 displayed 11 amino acid differences in antigenic epitopes (7-1 and 7-2) compared with RotaTeq G4 (**Figure 3**). The G9 strains showed four amino acid differences at positions 87, 100, 242, and 221 compared with the Rotasill G9 and Rotavac G9 strains, which mapped to the three (7-1a, 7-1b, and 7-2) neutralizing antigen epitopes. These G9 strains showed high similarity to the VP7 protein of Rotavac G9 and Rotasill G9 (**Table 2**). Furthermore, potential N-linked glycosylation sites at positions 69–71 were found in all 20 G9 strains.

### Comparison of VP4 neutralizing epitopes with vaccine strains

**Figure 4:**
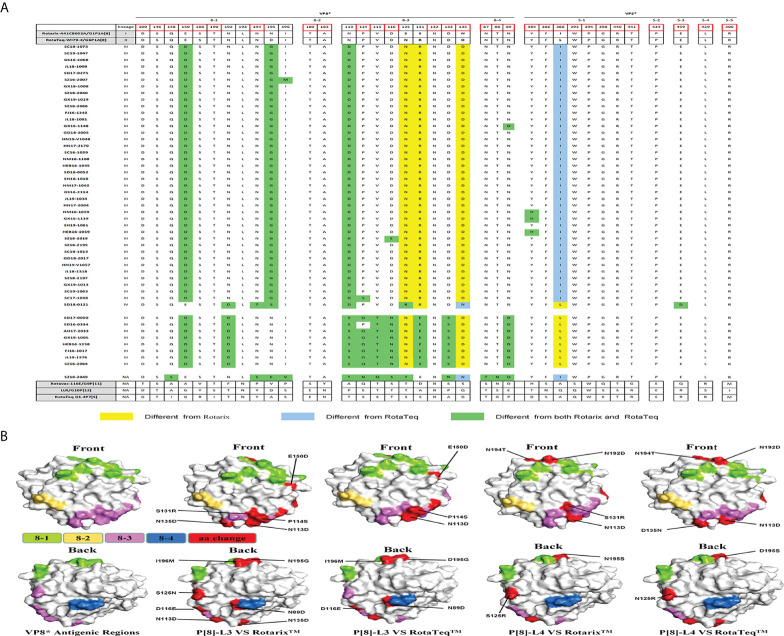
**(A)** Alignment of the antigenic epitopes in VP4 of RVA strains circulating in China with those in Rotarix, RotaTeq, and other vaccines. Red box, the site involved in neutralization escape. **(B)** Surface representation of the VP8* monomer (PDB 1KQR). Upper and lower images are of the front and rear, respectively, of VP8*. Antigenic epitopes are shown in chartreuse (8-1), yellow-orange (8-2), and violet (8-3). Red surface-exposed residues indicate differences between circulating and vaccine RVA strains.

There are four surface-exposed antigenic epitopes (8-1 to 8-4) in VP8* and five in VP5* of VP4. In this study, more mutations were found in VP8* than VP5* between P[4], P[6], or P[8] and the vaccine strains (**Figure 4**). Marked sequence variability in antigenic epitopes was observed between P[8] lineage III (P[8]-L3) and P[8] lineage IV (P[8]-L4) strains (**Figure 4A**). P[8] lineage III showed greater antigenic site differences with Rotarix P[8] than with RotaTeqP[8] (six or seven vs. four or five mutations). In addition, P[8] lineage IV showed eight and seven amino acid differences in antigenic epitopes compared with Rotarix P[8] and RotaTeqP[8], respectively (**Figures 4A, B**).

P[6] strain (SZ18-2049) showed 13 amino acid differences at antigenic sites compared with P[8] of Rotarix and 15 compared with P[8] of RotaTeq. Compared with P[8] of the Rotarix and RotaTeq strains, P[4] had 11 or 12 and eight or nine neutralizing antigenic site mutations, respectively, more of which were in VP8* (8-1, 8-3, and 8-5) than in VP5* (5-1) (**Figure 4A**). Interestingly, site N132 in region 8-3 was conserved among all circulating and vaccine strains.

## Discussion

Live-attenuated RVA vaccines have been introduced in more than 100 countries, contributing to herd immunity (Zeller et al., 2012; Ben Hadj Fredj et al., 2013). In countries in which an RVA vaccine was included in the national immunization plan (NIP), the number of hospitalizations due to rotavirus infection has decreased markedly (Cates et al., 2022). However, the RVA vaccine was more effective against severe rotavirus disease in high-income countries (80%–90%) than in low-to-middle-income countries (40%–70%) (Cates et al., 2022). Rotavirus vaccination does not affect the natural turnover of rotavirus strains. However, the fact that v were no longer predominant after RotaTeq and Rotarix introduction (Patel et al., 2011), vaccination is important for surveillance of circulating rotavirus strains. VP7 and VP4 are the most important rotavirus neutralization antigens, and analysis of their antigenic differences between circulating and vaccine strains is warranted.

In 2001, the predominant rotavirus strains shifted from G1 to G3 in China; after 2012, G3 was replaced by G9. The predominant P genotype has been P[8] for the past 20 years. In fact, from January 2016 to December 2019, 5147 samples were genotyped by PCR and sequencing. G9P[8] was most predominant, followed by G3P[8], G2P[4], G1P[8], G2P[4], and G4P[6] (unpublished data from the National Viral Diarrhea Surveillance Network of China). On the basis of preliminary phylogenetic analyses, we selected 50 strains representative of the circulating rotavirus genotypes and investigated the extent of their prevalence (years and regions) in mainland China. The two most widely used vaccines, Rotarix and RotaTeq, were included in the phylogenetic and neutralization epitope analyses of the VP7 and VP4 genes. Furthermore, Rotasill and Rotavac (G9 genotype) were included in the analysis of VP7 of G9. LLR (G10P[15] genotype), a completely attenuated strain from sheep, obtained marketing approval in 2001 in China. Because it has marked sequence differences in VP7 and VP4 compared with prevalent human strains, it was not analyzed further.

G1 RVA strains are generally of the P[8] genotype; therefore, both RotaTeq and Rotarix (which have G1 and P[8]) can provide homotypic protection against G1and P[8] strains. However, the VP7 epitopes of the prevalent G1 RVA strains in this study had N94S, which is associated with immune escape (Taniguchi et al., 1988). G2 RVA strains, which are uncommon in China, are generally associated with the P[4] genotypes. Compared with G2 of RotaTeq, only minor mutations were found in antigenic epitopes in the 7-1a and 7-1b regions of G2 strains. Compared with other G genotype strains, G3 strains did not possess more mutations but rather additional glycosylation site mutations, N238 (7-1b) and N145 (7-2), which are absent from G3 vaccine strains. Combined with previous reports and VP7 structural models, the immunogenicity of the 7-1a epitope could also be affected by glycosylation of residue N238 (Ben Hadj Fredj et al., 2013). K238N reduced neutralization of animal RVA strains (Ciarlet et al., 1994; Ciarlet et al., 1997). Therefore, this glycosylation site mutation may explain the antigenicity difference compared with G3 vaccine strains.

Analysis of the VP7 gene of G9 revealed that three later strains (GX19-1013, SZ18-2197, and SC19-1063) in G9 lineage III-d clustered with earlier Chinese strains (E1545), and other G9 lineage VI-e strains clustered with newly reported Chinese strains in GenBank. Similar findings were reported by a study in Jiangsu Province, China (Wang et al., 2015). However, in Africa and Belgium, G9 strains independently clustered in G9 lineage VI or III (Zeller et al., 2012; Xu et al., 2018; Manouana et al., 2021). Moreover, the 20 G9 strains in this study had potential N-linked glycosylation sites (positions 69–71), which are absent from VP7 of Rotasill and Rotavac. In addition, these glycosylation sites are rare in field RVA strains. Glycosylation determines viral immunogenicity by modulating virus receptor binding or masking antigenic sites, as in SARS-CoV-2 (Petrovic et al., 2021). Further studies should address whether these glycosylation sites alter the antigenicity of VP7 (Zeller et al., 2012; Harastani et al., 2020)

The G4 and P[6] genotypes in this study displayed marked intra-genotypic variety and high homology to porcine strains, indicating spillover of G4 and P[6] from pigs to humans in China. Indeed, P[6] is a widespread zoonotic RVA genotype in developing countries (Malasao et al., 2018). Consequently, P[6] is likely to spread between human and animals as a result of poor sanitation and constant contact with livestock. No approved RVA vaccine contains a P[6] or P[4] genotype strain. Partial heterotypic protection against P[4] and P[6] RVA is provided by the approved live-attenuated vaccines. However, it is difficult to compare the antigenic epitopes of P[6] or P[4] genotypes with those of vaccine strains.

P[8] is the most common rotavirus P genotype worldwide. Rotateq, Rotarix, and Rotavac with the P[8] genotype provide homotypic protection against P[8] strains. Consistent with reports from other countries, P[8] strains in China are mainly of P[8] lineage III (Zeller et al., 2012; Sadiq and Bostan, 2020). Interestingly, strain SD18-0121, the sole exemplar of P[8] lineage IV, showed low amino acid sequence similarity with the P[8] of vaccine strains, as is evident in the structure of VP4. VP8* is the location of most antigen mutations between vaccine and circulating strains. However, the P[8] RVA strains in this study had few amino acid changes in their VP8* epitopes. Four strains (NM16-1059, GX16-1119, HEB16-1059, and SD18-0121) showed amino acid mutations in VP5*. These amino acid differences in antigenic epitopes warrant further investigation of their effects on vaccine efficacy.

RVA vaccination has significantly reduced the burden of RVA disease among children worldwide, especially in developing countries. RotaTeq and LLR are approved in mainland China but are not included in the NIP. A study in six provinces of China in 2018 showed a low rate of LLR rotavirus vaccination and late vaccination age, which may be improved by addition of an RVA vaccine to the NIP. Compared with the vaccine strains, the amino acid differences in the VP7 and VP4 antigenic epitopes of Chinese strains may reduce vaccine effectiveness, necessitating further research on these epitopes. In addition, efforts to accelerate the development of new RVA vaccines, including reverse genetics, are warranted.

## Data availability statement

The datasets presented in this study can be found in online repositories. The names of the repository/repositories and accession number(s) can be found in the article/supplementary material.

## Ethics statement

Ethical review and approval was not required for the study on human participants in accordance with the local legislation and institutional requirements. Written informed consent from the participants’ legal guardian/next of kin was not required to participate in this study in accordance with the national legislation and the institutional requirements.

## Author contributions

TM, DL, and ZD were involved in the design of this study. MWW and MXW performed RNA extraction, sequencing, and sequences classification. TM, JW, YM, and LL performed molecular and phylogenetic analyses. TM and XS performed molecular structure diagram analyses. XL, HL, and QZ participated in sample collection and data analysis. TM wrote the manuscript. ZD was responsible for the critical revision of the manuscript. All authors contributed to the article and approved the submitted version.

## Funding

This work was supported by National Natural Science Foundation of China (grant no. 21934005).

## Acknowledgments

The authors thank the National Viral Diarrhea Surveillance Network in China.

## Conflict of interest

The authors declare that the research was conducted in the absence of any commercial or financial relationships that could be construed as a potential conflict of interest.

## Publisher’s note

All claims expressed in this article are solely those of the authors and do not necessarily represent those of their affiliated organizations, or those of the publisher, the editors and the reviewers. Any product that may be evaluated in this article, or claim that may be made by its manufacturer, is not guaranteed or endorsed by the publisher.
