# Corrigendum: Phylogenetic analysis of the viral proteins VP4/VP7 of circulating human rotavirus strains in China from 2016 to 2019 and comparison of their antigenic epitopes with those of vaccine strains

**DOI:** 10.3389/fcimb.2024.1455093

**Published:** 2024-08-12

**Authors:** Tongyao Mao, Mengxuan Wang, Jindong Wang, Yalin Ma, Xiafei Liu, Mingwen Wang, Xiaoman Sun, Lili Li, Huiying Li, Qing Zhang, Dandi Li, Zhaojun Duan

**Affiliations:** ^1^ NHC Key Laboratory for Medical Virology and Viral Diseases, National Institute for Viral Disease Control and Prevention, Chinese Center for Disease Control and Prevention, Beijing, China; ^2^ Department of Medical Microbiology, Weifang Medical University, Weifang, China; ^3^ School of Public Health, Gansu University of Chinese Medicine, Lanzhou, China

**Keywords:** rotavirus vaccines, antigenic epitopes, diarrhea, VP7, VP4, China

## Incorrect affiliation

In the published article, there was an error in affiliation(s) **1**. Instead of **Key Laboratory for Medical Virology and Viral Diseases, National Institute for Viral Disease Control and Prevention, National Health Commission of the Peoples Republic of China, Beijing, China**, it should be **NHC Key Laboratory for Medical Virology and Viral Diseases, National Institute for Viral Disease Control and Prevention, Chinese Center for Disease Control and Prevention, Beijing, China**.

The authors apologize for this error and state that this does not change the scientific conclusions of the article in any way. The original article has been updated.

